# Taxonomic Classification for Living Organisms Using Convolutional Neural Networks

**DOI:** 10.3390/genes8110326

**Published:** 2017-11-17

**Authors:** Saed Khawaldeh, Usama Pervaiz, Mohammed Elsharnoby, Alaa Eddin Alchalabi, Nayel Al-Zubi

**Affiliations:** 1 Erasmus+ Joint Master Program in Medical Imaging and Applications, University of Burgundy, 21000 Dijon, France; 12beeupervaiz@seecs.edu.pk; 2 Erasmus+ Joint Master Program in Medical Imaging and Applications, UNICLAM, 03043 Cassino FR, Italy; 3 Erasmus+ Joint Master Program in Medical Imaging and Applications, University of Girona, 17004 Girona, Spain; 4 Graduate School of Natural and Applied Sciences, Istanbul Sehir University, 34865 Kartal/İstanbul, Turkey; mohamedelsharnouby@std.sehir.edu.tr (M.E.); alaaalchalabi@std.sehir.edu.tr (A.E.A.); 5 Department of Computer Engineering, Al-Balqa’ Applied University, 19117 Al-Salt, Jordan; nael.alzubi@bau.edu.jo; 6 Department of Electrical Engineering and Automation, Aalto University, 02150 Espoo, Finland

**Keywords:** DNA, genes, taxonomic classification, convolutional neural networks, encoding

## Abstract

Taxonomic classification has a wide-range of applications such as finding out more about evolutionary history. Compared to the estimated number of organisms that nature harbors, humanity does not have a thorough comprehension of to which specific classes they belong. The classification of living organisms can be done in many machine learning techniques. However, in this study, this is performed using convolutional neural networks. Moreover, a DNA encoding technique is incorporated in the algorithm to increase performance and avoid misclassifications. The algorithm proposed outperformed the state of the art algorithms in terms of accuracy and sensitivity, which illustrates a high potential for using it in many other applications in genome analysis.

## 1. Introduction

Taxonomy is the biological area that recognizes, reports, classifies and names the discovered and undiscovered species and other taxa. Recently, species’ taxonomy became a difficult task, as it requires full integration of new theories, methods and data from all areas that study the origin, limits and evolution of species [1,2]. Classification is one of the data mining techniques that is used to categorize samples into different classes based on supervised learning [3].

**Figure 1:**
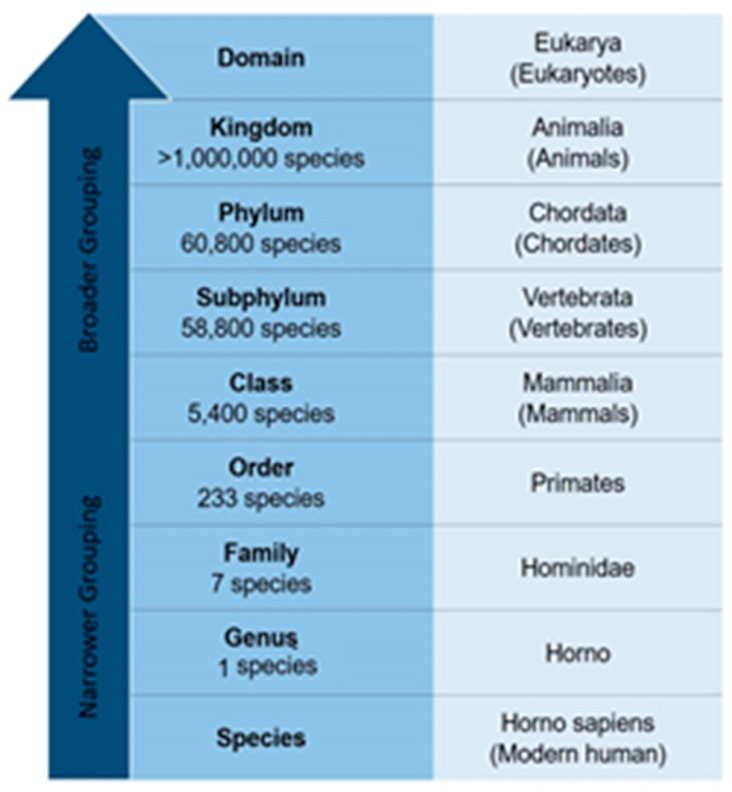
Taxonomy Hierarchy of Modern Human.

Taxonomic classification is a hierarchical system used to categorize organisms to the species level, as is shown in Figure 1. The higher classifications are by domain and kingdom levels; however, the deepest and the most detailed classification is to genus and species levels. The hierarchical levels in between include phylum, subphylum, class, family and order [4]. DNA classification can alter humans’ understanding of the relationships between the different species, help understanding how the classification changes over years and boost the understanding of how the entire kingdom of life system works [5]. Animal and plant classification was based only on morphological characteristics since the beginning of living creatures’ classifications [6]. However, due to the development of DNA sequencing technology and the availability of high computational power—multicore central processing units (CPUs) and graphical processing units (GPUs)—required to process DNA sequences and interpret useful information about them, a radical shift to DNA-based classification started taking place [7].

Because of the shift to the DNA-based classification and analysis, various genome analysis tools were developed by researchers for DNA, RNA and protein sequence analysis and processing. Some of these tools incorporated traditional machine learning techniques such as support vector machine (SVM), neural networks and *k*-nearest neighbor (KNN). In [8], the authors developed a Python package called “repDNA” to produce the features representing physicochemical properties and the sequence-order effects of DNAs and nucleotides. Fifteen types of features built from DNA sequences can be calculated using the repDNA package. These computed features can be categorized into three different categories: nucleic acid composition, autocorrelation and pseudo-nucleotide composition. The repDNA internal algorithms were evaluated by generating many test DNA sequences, then the generated output was compared with the ground truths of these sequences to ensure having an error-free implementation. The package incorporates several traditional machine learning algorithms to be used with the generated features. In [9], the authors developed a web server called “repRNA”, which can produce eleven modes of feature vectors. It also enables users to choose any set of features out of 22 built-in physicochemical properties. Moreover, repRNA gives the option to visualize the generated feature vectors through a built-in visualizer incorporated in the web server. repRNA makes it easy for users to perform any in-depth genome analysis as it can easily incorporate machine learning algorithms such as SVM and KNN, which were not capable of handling anything except vectors back then. In [10], the authors developed a Python package called “Pse-Analysis”, which can automatically perform feature extraction and selection, parameter tuning, model training, cross-validation and evaluation for any biological sequences. The training in Pse-Analysis uses the SVM machine learning model; also, the package provides a powerful multi-core CPU implementation, which efficiently decreases the running time compared to the one-core CPU implantation. In [11], the authors developed a web server called “Pse-in-One”, which can reproduce almost every possible feature vector based on a set of properties defined by the user for DNA, RNA and protein sequences. The web server provides an easy way to combine the vectors with different machine learning techniques such as SVM and neural networks. Pse-in-One, the first developed platform of its kind, includes 148, 22 and 547 built-in physicochemical properties, which can be defined by users before generating the DNA, RNA and protein sequences.

The ensemble learning approach is a common method used to acquire better performance through using multiple learning techniques. In [12], the authors multiply used three random forest classifiers as an ensemble classifier to find out the location of the DNase I Hypersensitive Site (DHS) in the human genome, which gives critical information to determine the functionality of noncoding genomic regions. In [13], the authors were interested in studying the mechanism of meiotic recombination and genome evolution; therefore, they developed a classifier through combining various modes of Pseudo *K*-tuple Nucleotide Composition (PseKNC) and the mode of Dinucleotide-based Auto-Cross-Covariance (DACC) to ensemble a clustering technique. Both algorithms in [12,13] outperformed the state of the art algorithms such as [14,15,16,17], respectively, which illustrates the strength of ensemble learning solutions.

Inspired by the success of applying some popular text classification methods to the barcode and DNA analysis domain such as the naive Bayes method in [18], probabilistic topic modelling in [19], character-based methods in [20] and its counterparts in Natural Language Processing (NLP) in [21,22], convolutional neural networks (CNNs) started to be considered as a model for DNA barcode analysis, which led to integrating them with several genome analysis tools and platforms. In [23], the authors wanted to provide an in-depth understanding of how to select the CNN architecture that matches the task that they wanted to implement. Furthermore, they have performed a systematic exploration of the performance of different CNN architectures for the fundamental genomic task of characterizing the binding affinity of transcription factors to the DNA sequence in 690 different ChIP-seq experiments. Results showed that for both tasks performed (motif discovery and motif occupancy), classification performance increases with increasing the number of convolution kernels. They observed as well that the use of local pooling or more convolutional layers has only a small, even sometimes a negative, effect on the performance. 

In [24], the authors introduced a new method to classify DNA sequences by considering them as text. They used the CNN technique to perfume the classification. The concept “one-hot vector”, which saves the position records of each nucleotide in DNA sequences, was used. By applying the same representation technique of text, they obtained a two-dimensional numerical matrix, which contained information about the specific position of each nucleotide in the sequence. Then, this matrix was finally used as input to the CNN model to perform the classification of the DNA sequences into different classes. To show the efficiency of their classification method, they selected the 10 datasets that were used in [25], then they applied the proposed algorithm on them to compare the results obtained by their algorithm with the results obtained by other methods such as using *n*-mer features as the representation of sequences and SVM as the classifier. The lowest improvement they obtained was nearly 1% for accuracy, and the highest improvement was over 6% for accuracy. These improvements were quite high in comparison with other approaches such as finding good representations for sequences or feature selection that were applied before. Results showed that features extracted by CNNs were very useful for the classifier to classify sequences into their different classes. In [26], the authors investigated how good they can represent biological functions using only the examination of a raw sequence. To perform their experiment, they used a large corpus of labelled protein sequences, then they learnt dense vector representations for amino acid sequences using the co-occurrence statistics of short fragments. Afterwards, using vector representation, they experimented with several neural network architectures to train the proposed classifiers for protein family identification. The proposed method illustrated a good performance for multi-class prediction with 589 protein family classes. Their results showed that neural network classifiers give better performance than the tuned SVM baseline on the same UniProt dataset; particularly Gated Recurrent neural networks (GRU) outperformed their SVM baseline by almost 7%. In comparison to the SVM in [27], which trained many single-class classifiers, the neural network (NN) architectures of the multi-class protein performed well relatively, even if being more complicated. In [28], the authors investigated the reliability of Frequency Chaos Game sequence Representation (FCGR) for the classification of genomic sequences. They chose a CNN to solve the classification problem, and the obtained results were very good, especially for the full-length sequences, which were recognized with a very high accuracy. In [29], the authors presented a new method for the prediction of protein disorders based on sequence information. Their approach used deep networks to make predictions. In a 10-fold cross-validation procedure on a dataset composed of 723 proteins, the method achieved an accuracy of 82% and an area under the receiver operating characteristic (ROC) curve of 90%. In [30], the authors used Bidirectional Long Short-Term Memory (BLSTM) to look for powerful features from pseudo-proteins; moreover, they proposed the “ProDec-BLSTM” classifier, which contains an input layer, BLSTM, a time distributed layer and, finally, an output layer. This designed architecture could automatically extract features through using the BLSTM and the time distributed dense layer. The ProDec-BLSTM classifier outperformed methods reported in [31,32,33,34,35,36,37] in terms of mean ROC and mean ROC50 scores, which were 96.9% and 84.9%, respectively. In [38], the authors proposed a classifier based on deep learning for the estimation of human protein subcellular localization. For the implementation of the proposed algorithm, the authors utilized GPUs and CUDA software (nVidia Corp., Santa Clara, CA, USA) to tune the designed network parameters and effectively train them. Moreover, it was reported that the used architecture could automatically learn the high-level features that represent the human proteins by going over the polynomial relations between the sparse subcellular locations. The dataset used in their study contained 13,978 samples belonging to 10 different classes representing the subcellular locations. The algorithm acquired a total accuracy of 37.4% of correct predictions for the 10 classes, which means that it could correctly predict 5226 samples out of the total number of samples available in the dataset.

In this work, we propose a convolutional neural network-based approach to classify DNA sequences of living organisms. The proposed methodology achieved the highest classification accuracy compared to the state of the art techniques presented in the literature. We propose an efficient solution for the low performance encountered when analyzing the DNA data without involving any pre-processing step. The proposed solution includes encoding the DNA sequences to reduce similarities between the different sequences that cause misclassification in the test set.

### Problem Definition

Classifying each DNA sequence to which taxonomy (class, order, family, etc.) it belongs is the problem this work addresses. Figure 1 presents the different levels in the taxonomy hierarchy. The modern human species is taken as an example to show the names of the different levels to which modern humans belong. DNA sequences are represented as a string that contains {’A’, ’C’, ’G’, ’T’, ’-’} characters. More specifically, this paper presents a deep learning-based approach to identify the taxonomy based on DNA sequences collected from different species. CNN is the machine learning technique used to tackle the taxonomic classification problem in this work. The inputs of the CNNs are the species’ DNA sequence fragments, while the output is the class label to which the DNA sequence fragment belongs.

**Figure 2:**
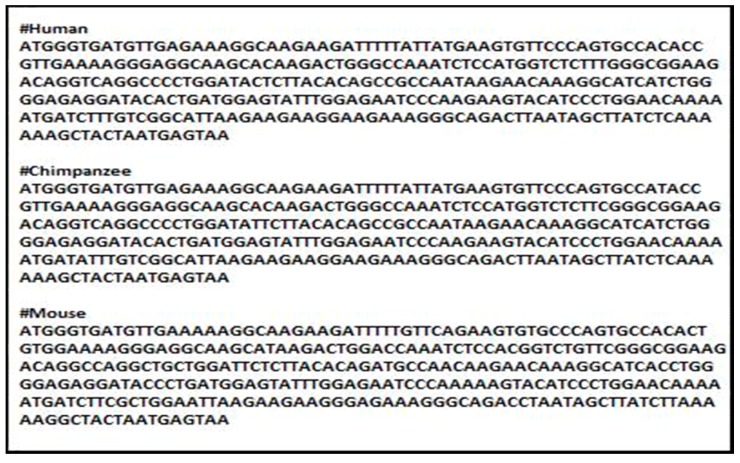
Samples represent DNA sequences for *Cytochrome C* in human, chimpanzee and mouse.

Figure 2 shows an example that illustrates the difference between the DNA sequences for *Cytochrome C* in three different species: human, chimpanzee and mouse, respectively.

## 2. Materials and Methods

### 2.1. Methodology

**Figure 3:**
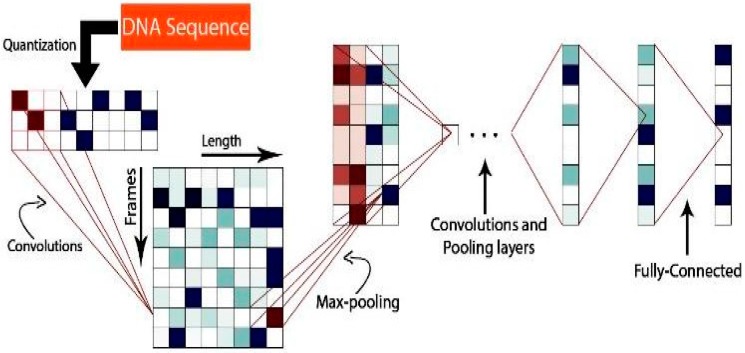
Illustration of the convoluted neural network (CNN) model used.

Figure 3 shows a modified version of the CNNs model in [20] that was designed to understand text using deep learning. In the understanding text problem, the inputs were quantized characters; however, for taxonomic classification, the inputs are DNA sequences, which only have five characters {’A’, ’C’, ’G’, ’T’, ’-’}. In the understanding text problem, the outputs were the abstract properties of the text; however, for taxonomic classification, the output is the taxonomy label.

The model’s main component is the temporal convolutional module, which computes the 1D convolution between the input *g*(*x*) and the kernel *f*(*x*) to generate the output *h*(*y*), as is shown in Equation (1); where *c* represents the offset constant.

(1) $$h\left(y\right)=\sum_{x=1}^{k}f\left(x\right)\times g\left(y\times d-x+c\right)$$

The model uses the same spatial max-pooling module that is used in computer vision, as in [39]; however, it is in 1D in our case; where *g*(*x*) represents the input function and *h*(*y*) represents the max-pooling function. See Equation (2) below.

(2) $$h\left(y\right)=max_{x=1\;}^{k}g\left(y\times d-x+c\right)$$

### 2.2. Implementation and Experimentation

The Torch7 platform was used to implement the CNN model for this work. The reason for selecting Torch7 is because it provides the maximum possible flexibility and speed in building the scientific algorithms including the different machine learning techniques [40]. Crepe is the name of the code that was implemented using Torch7 for text classification from the character-level dataset using CNNs [41], and it has been used to produce the results in [20]. Crepe contains the following components:

1. Data preprocessing scripts that can be used to convert csv format to a Torch 7 binary format (t7b) that can be used by the training program directly: This component contains a tool called csv2t7b.lua; it is used to convert the datasets of csv format to t7b format, which can be fed to Crepe’s training component. This data preprocessing component contains two command-line parameters as shown in Table A1 in the Appendix A. The dataset t7b format can be loaded using the regular torch calls. The variable train has three members as shown in Table A2 in the Appendix A.
2. The training program is a code used to process the data representing the DNA sequences: Originally, this code processed text and classified it into different classes representing the topic to which the text belongs; however, for the DNA taxonomic classification, we modified it to process DNA sequences. The reason for pursuing a nearly similar approach for processing both text and DNA data is that the two types of data are highly similar. The training subsets are fed first to the training component program in Crepe to train the classifier. Then, the model accuracy is evaluated on the remaining subsets, which were spared for testing. Table A3 shows the different training programs in Crepe.

The data used in this project were obtained from the Barcode of Life Data Systems (BOLD) Web Services, which provides the ability to download specimen, sequence, and trace data from the Public Data Portal [42]. Nine orders have been selected and downloaded from the BOLD Web Services website. Order names were replaced with number labels for simplicity. Table 1 below shows the nine classes available in the dataset that we used in our experiments.

This dataset obtained from BOLD Web Services was merged, then transformed from *fas* format (the original format of the data) to *csv* format (the format that we feed to our model), then it was divided into two subsets:

1. Training subset: including approximately 78.72% of the original dataset.
2. Test subset: including approximately 21.28% of the original dataset (11.45% was used for cross-validation and 9.83% for testing).

The two subsets were transformed from *csv* format to t7b format using the Crepe data processing script described above. In the total dataset formed, 31,068 DNA sequences were available; each sequence has its corresponding class label provided by the BOLD website. In the training subset, we had 24,455 samples, while we had 6613 samples in the test subset (3558 samples for cross-validation and 3055 samples for testing). The samples in each data division were randomly selected from the main dataset, which contained the 31,068 DNA sequences.

Two types of experiments were performed after preparing the different data subsets as explained earlier.

- Experiment 1: feeding the DNA sequences to our CNN model without making any changes to them. Each DNA sequence is represented by a string, containing {’A’, ’C’, ’G’, ’T’, ’-’} characters and a label that shows to which class this DNA sequence belongs.
- Experiment 2: Encoding the DNA sequences by creating unique labels. Each unique label represents three characters in the original DNA sequence. A dictionary was generated to guide encoding the DNA sequences. The purpose of this encoding process is to ensure having a dataset similar in complexity and variety to the one used in the text classification problem.

**Figure 4:**
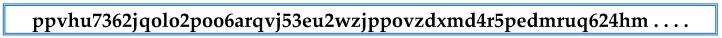
Encoded DNA sample from Class 9.

Applying encoding is expected to help the CNN model perform better as more differences are introduced between the various DNA sequences in the dataset. Figure 4 below shows the beginning of a randomly-selected sample from an encoded DNA sequence that belongs to the “Valvatida” class. More details about the “3-1” encoding used in this project are available in the Supplementary Materials.

## 3. Results and Discussion

In Experiment 1, our CNN model did not learn well; thus, the accuracy obtained on the test subsets was very low (around 40%). The reason for this poor performance is that the model was originally designed for the text classification problem, so the input was expected to be a set of characters that includes letters, number, and special characters. As for our dataset on which we ran our CNN model, each sample had only {’A’, ’C’, ’G’, ’T’, ’-’} characters, so the CNN model did not find many differences between the different DNA samples belonging to different classes. This led to many misclassifications, causing a very poor performance.

**Figure 5:**
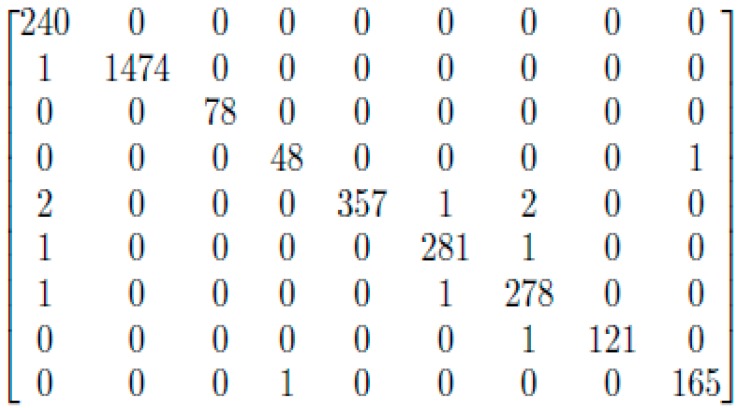
Confusion Matrix.

**Figure A1:**
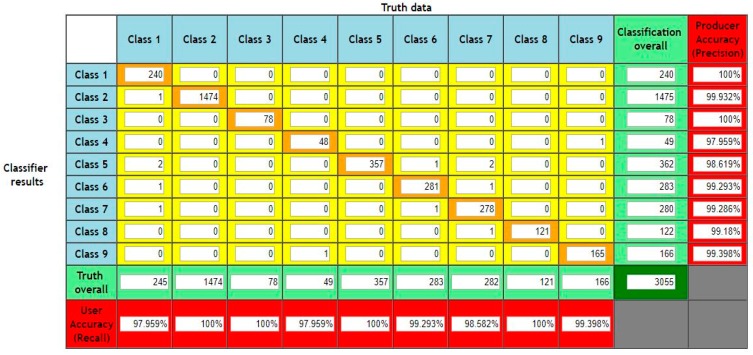
Confusion matrix, precision, and recall values for all classes.

In Experiment 2, the “3-1” encoding was performed on our dataset. Then, we trained the CNN model on the new encoded training subset and validated and tested it on the encoded test subset. The data division for both experiments was identical to what was explained previously. However, this time, the results obtained showed a significant improvement (accuracy refers to the closeness of a measured value to a standard or known value; also, it is defined as the number of true predictions divided by the total number of predictions; in our case, the overall accuracy was 99.574%). The recall (or sensitivity) is defined as the fraction of relevant samples that have been retrieved over all relevant ones; while the precision is defined as the fraction of relevant samples among the retrieved ones. In our case, the average recall and precision for all the classes were 99.24% and 99.30%, respectively. All the values of the performance measures were calculated using [43] based on the confusion matrix shown in Figure 5 below. For more details about the performance measures, see Figure A1 in the Appendix A.

The results above show a very high potential for using our approach, which incorporates CNN model and DNA encoding, for the various applications in the DNA classification domain. The high values of accuracy, recall and precision that our approach reported outperform the state of the art approaches reported in the literature. For instance, in [38], the authors used deep learning for human protein subcellular localization; however, they did not perform encoding, and they obtained an overall accuracy of 37% for the 10-class problem, which they have addressed in their work. In addition, the authors in [30] did not use accuracy as a performance measure to evaluate the performance of BLSTM; nevertheless, they reported mean ROC and ROC50 values of 96.9% and 84.9%, respectively, which is still considered lower than the archived performance measures in our proposal. Several other papers in the literature addressed also genome analysis, such as [8,9,10,11]; nevertheless, none of them incorporated deep learning nor DNA encoding, but instead, they incorporated traditional machine learning techniques such as *k*-means, SVM and traditional neural networks. Additionally, they were primarily for DNA, RNA and protein feature vector generation and processing, unlike our proposal, which integrates deep learning and encoding for DNA sequence classification. Even though the ensemble learning approach in [12,13] outperformed some of the state of the art algorithms by obtaining an overall accuracy of 86.14% and 82.65%, respectively, still, our algorithm has a higher accuracy than them.

## 4. Conclusions

To the best of our knowledge, this study is the first one utilizing the CNN model designed for text classification for taxonomic classification of living organisms. The DNA sequences were first encoded, then fed to the modified CNNs for training, then testing. The results obtained show a high potential in pursuing this approach to solve more complicated problems like classification of DNA sequences to deeper levels in taxonomy hierarchy. Performing more experiments while following a similar procedure to the one presented in this work, increasing the number of samples and classes in the dataset and changing the taxonomy level up/down surely will further help with obtaining a more comprehensive conclusion about the efficiency of the approach in tackling more complicated genomic analysis problems.

Additionally, our proposed algorithm can be considered to capture local conservation and sequence-order information from Position-Specific Scoring Matrices (PSSMs) [44], as the performance of the current state of the art machine learning techniques that address this problem is still unsatisfactory. Furthermore, it can be used to predict the protein methylation sites accurately, which is critical for finding out more about the molecular mechanisms undergoing methylation. Our algorithm will potentially help increase the prediction accuracy, as all other machine learning algorithms, such as [45], cannot completely avoid the accuracy limitation, which is reported in many state of the art algorithms in the literature. Finally, our algorithm can be considered also for finding the location of DHS in the human genome or studying the mechanism of meiotic recombination and genome evolution, which were addressed in [12,13].

## Figures and Tables

**Table 1:** Labels used for different classes in the dataset.

Order Name	Label
Chaetothyriomycetes	1
Diptera	2
Echinoida	3
Forcipulatida	4
Lepidoptera	5
Onygenales	6
Pezizomycetes	7
Scleractinia	8
Valvatida	9

## Supplementary Materials

The following are available online at www.mdpi.com/2073-4425/8/11/326/s1. Supplemental file 1: Python scripts that developed to encode the DNA sequences as reported in the paper. *DNAtoCharsMappers.pyc* and *encoder_3_1.pkl*.

## Appendix A

**Table A1:** Data preprocessing command-line parameters.

***input [file]***	This inputs the csv format file. The input file at this stage is in ***csv*** format where the entries of the first column of it are numbers indicating each class label, starting from 1 to ending with 9 (nine classes). Other columns are considered as text fields.
***output [file]***	This outputs the t7b format file. The output file at this stage is in torch-7-binary format (t7b). These generated files are directly used in training and validating datasets for the Crepe training program component.

**Table A2:** Members of the variable *train*.

***train.content***	A ***torch.ByteTensor*** that stores concatenated string data. Each string is ended with *NULL (0)*.
***train.index***	A ***lua*** table for which ***train.index[i]*** is a 2D ***torch.LongTensor***. The ***train.index[i][j][k]*** indicates the offset in ***train.content*** for the string in class *i*, *j*-th sample and *k*-th field.
***train.length***	A ***lua*** table for which ***train.length[i]*** is a 2D ***torch.LongTensor***. The ***train.length[i][j][k]*** indicates the length for the string in class *i*, *j*-th sample and *k*-th field. The length does not count the ending NULL.

**Table A3:** Training programs for Crepe.

***config.lua***	A unified file for all configurations for the dataset, model, trainer, tester and GUI
***data.lua***	Provides a Data class; both training and validating datasets are instances of this class
***main.lua***	The main driver program
***model.lua***	Provides a Model class; it handles model creation, randomization and transformations during training
***mui.lua***	Provides a Mui class; uses the Scroll class to draw an nn.Sequential model in Qt
***scroll.lua***	Provides a Scroll class that starts a scrollable Qt window to draw text or images
***scroll.ui***	A Qt designer UI file corresponding to the scrollable Qt window
***test.lua***	Provides a Test class; handles testing, giving you losses, errors and confusion matrices
***train.lua***	Provides a Train class; handles training with Stochastic Gradient Descent (SGD) and supports things like momentum and weight decay
